# Dynamic changes in amygdala and insula responses to the onset and offset of conditioned stimuli during threat learning

**DOI:** 10.1038/s41598-026-62910-8

**Published:** 2026-07-17

**Authors:** Olle Berggren, Gránit Kastrati, Fredrik Åhs, Jörgen Rosén

**Affiliations:** 1 Department of Clinical Neuroscience, Karolinska Institutet, Stockholm, Sweden; 2 Department of Education, Psychology and Social Work, Mid Sweden University, Östersund, Sweden

**Keywords:** Fear conditioning, fMRI, Partial reinforcement, Unconditioned stimulus omission, CS termination, Amygdala, Insula, Neuroscience, Psychology, Psychology

## Abstract

The amygdala and insula are central to learning how to anticipate and respond to threats, yet their precise roles during different phases of fear conditioning remain incompletely understood. In this study, we used functional magnetic resonance imaging (fMRI) to examine amygdala and insula responses in a large sample (*N* = 286) of adult twins during fear conditioning. Participants were exposed to two virtual characters: one (CS +) was paired with a mild electric shock (US) on 50% of acquisition trials (partial reinforcement), while the other (CS −) was never paired with the US. During habituation, neither the amygdala nor the insula discriminated between the cues. During acquisition, amygdala responses were strongest and most consistent at CS + offset on non-reinforced CS + trials (i.e., when the US was expected but omitted), whereas insula responses differentiated CS + from CS − at both CS onset and CS offset. Whole-brain analyses showed that CS differentiation at CS onset expanded from early engagement of anterior insula and temporal regions to broader networks as learning progressed. CS differentiation at CS offset (non-reinforced CS +  > CS −) emerged in posterior insula and extended to broader predominantly contralateral networks in later acquisition blocks. Voxel-wise pattern similarity analyses indicated high within-event similarity across acquisition blocks (onset-with-onset; offset-with-offset), but lower similarity between event types (onset vs offset) and between either CS-related event and the US. Together, these findings are consistent with partially distinct large-scale activation patterns across cue onset, omission-related cue offset, and US delivery during threat learning.

**Supplementary Information:**

The online version contains supplementary material available at 10.1038/s41598-026-62910-8.

## Introduction

Learning to respond to threats in the environment is essential for survival and is supported by evolutionarily conserved neural circuits. One of the most widely used paradigms for investigating how individuals learn to predict threats from environmental cues is fear conditioning. In this paradigm, a previously neutral cue becomes associated with an aversive event through experience, and once learning has occurred, this cue is referred to as the reinforced conditioned stimulus (CS +). Research in humans, mice, and rats has demonstrated that individuals begin to exhibit defensive responses to the onset of the CS + after repeated pairings with an aversive event, such as an electric shock (unconditioned stimulus, US)^1–3^.

Numerous studies in rodents have shown that neural activity in the lateral and central nuclei of the amygdala increases at the onset of the CS + following fear conditioning, supporting the idea that amygdala neurons are critical for learning to predict threats^4–6^. These findings have prompted many investigations into whether similar computational roles may be reflected in human neuroimaging measures. However, human fear conditioning studies using functional magnetic resonance imaging (fMRI) to measure amygdala activation during CS onset have produced mixed results. Some studies have not found greater amygdala activation to the CS + compared to a control cue (CS −)^7,8^, whereas other work has reported CS +  > CS − effects under some study designs and analysis choices^3,9^. As emphasized in meta-analytic work, heterogeneity in amygdala findings likely reflects differences in paradigm parameters (e.g., reinforcement schedule), modeling/analysis choices, and sample characteristics, rather than sample size alone^7^. However, few studies have investigated changes in amygdala reactivity to the CS + and CS − over the time course of conditioned learning (see Wen et al., 2022 for an exception).

Beyond the amygdala, the insula is a key region involved in fear conditioning^7,11^. The insula is implicated in interoceptive awareness and emotional processing, and neuroimaging studies have shown that it is activated during both the anticipation and experience of aversive stimuli^7,11^. During fear conditioning, the insula is thought to contribute to the subjective experience of threat and to the integration of bodily states with emotional responses^17^. Recent research indicates that both the amygdala and insula can show increased activation to conditioned stimuli associated with threat, with the insula often implicated in processing anticipated aversive outcomes and the salience of the CS + ^7,11^.

The amygdala is also involved in orchestrating responses to the actual occurrence of aversive events (US). Nociceptive stimulation during fear conditioning is associated with increased amygdala activity^12^. It is therefore hypothesized that during fear conditioning, the amygdala participates both in predicting threat at CS + onset and in responding to the predicted threat (i.e., the US) at the time it is expected to occur. In delay fear conditioning, the expected moment of the US typically coincides with CS + offset. Some studies have investigated amygdala responses at the time when a US is expected, such as those by LaBar and Phelps^13^. In addition, several studies have examined responses at the offset of non-reinforced CS + trials in partially reinforced designs, which permit assessment of neural activity at CS + termination when the US is expected but omitted^14,15^. However, these studies have generally not directly compared neural responses at CS onset and at CS offset (on non-reinforced CS + trials) within the same dataset^16^, nor have they systematically examined how amygdala responses evolve across learning from habituation (no US presentations) to late acquisition, particularly at CS + offset.

Influential psychological theories suggest that the same brain regions that respond to aversive events also learn to predict them as sensory inputs converge in these regions^17–19^. The same regions are also thought to respond to prediction errors when predicted events are omitted. This has influenced the analyses of neuroimaging studies of prediction error that fits models to specific regions in the brain^20,21^, assuming the same region that serve prediction error will also show changes in activity to cues predicting aversive outcomes. In line with these ideas, previous fMRI studies comparing predicted, but omitted, aversive events to delivered aversive events have drawn conclusions that brain activation patterns are largely overlapping^14,16^. However, previous studies of US omission did not quantify the similarity in evoked brain responses between onset of CS + (anticipation/prediction of US), offset of unreinforced CS + (US omission) and US delivery. We sought to provide evidence for the idea that the same regions that anticipate US delivery (CS + onset) also responded to US omissions (unreinforced CS + offset) and to US delivery.

Despite extensive research on fear conditioning, important gaps remain in our understanding of how the amygdala and insula contribute to threat-related processing at different moments within a conditioning trial. Most human neuroimaging studies have emphasized cue-period responding (i.e., responses time-locked to CS onset), whereas fewer studies have directly examined neural responses at the time the US is expected to occur (e.g., at CS + offset on non-reinforced CS + trials in partially reinforced designs). Furthermore, the temporal dynamics of these responses, how they evolve from habituation to later acquisition, have not been systematically investigated, particularly for CS + offset responses. By directly comparing amygdala and insula responses to CS onset and CS offset across different phases of fear learning, this study seeks to clarify the dynamic roles of these regions in threat anticipation and responses at the expected moment of threat. We also examined block-wise changes in whole-brain CS differentiation at CS onset and CS offset. Finally, we quantified the similarity of whole-brain activation patterns across event types (CS onset, CS offset on non-reinforced CS + trials, and US delivery) by correlating voxel-wise contrast maps, to assess whether evoked activity patterns were similar across these event types as suggested by some empirical and theoretical accounts^14,16^. The number of participants in our study (*N* = 286) was considerably larger than in many prior studies, which permitted us to investigate effects in the amygdala, a region that can be challenging to study with fMRI due to susceptibility-related signal loss and noise. These insights are crucial for advancing our understanding of the neural mechanisms underlying conditioned threat processing over the course of fear learning.

## Results

### Amygdala ROI parameter estimates

**Fig. 1:**
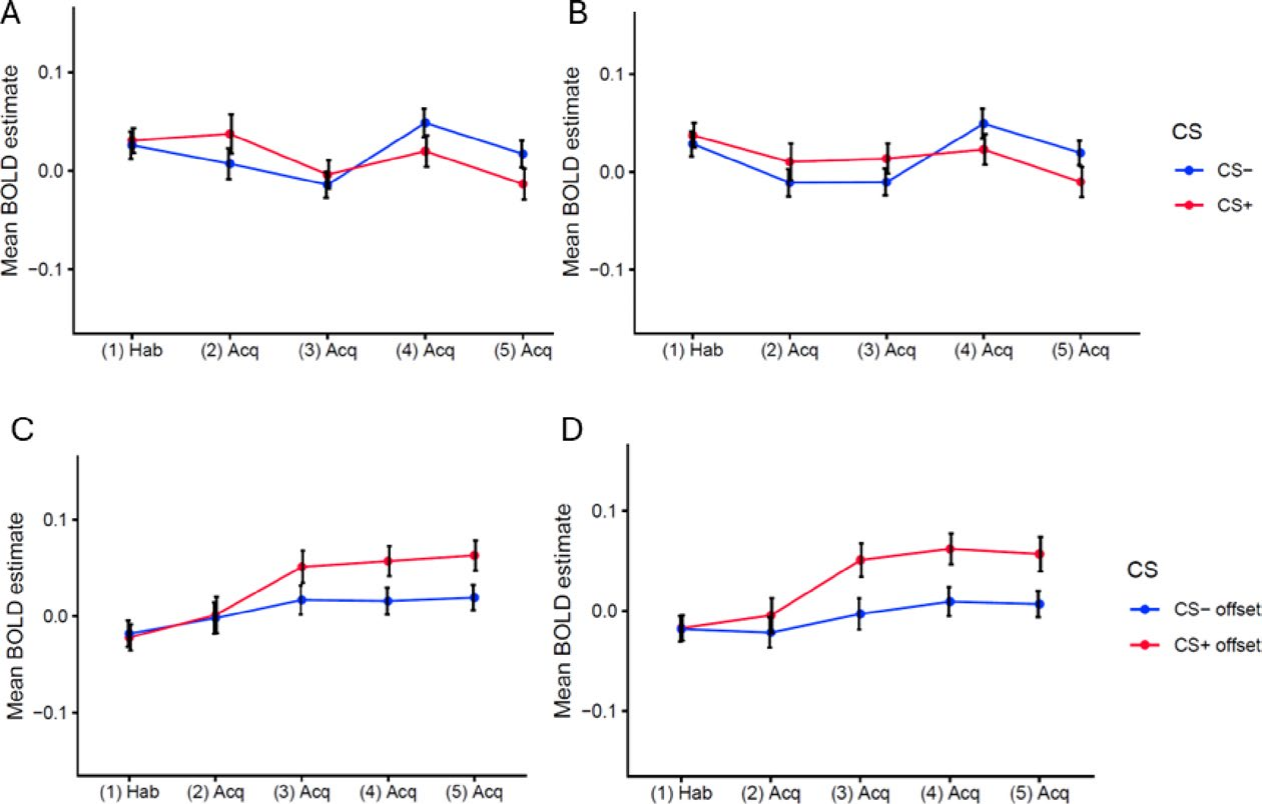
Amygdala ROI estimates to the onset of CS + and CS − presentations are shown for the (**A**) left and (**B**) right amygdala. Amygdala responses to the offset of CS + and CS− presentations are displayed for the (**C**) left and (**D**) right amygdala. Trials 1–4 are labeled as Hab, whereas trials 5–8, 9–12, 13–16, and 17–20 are labeled Acq 2–5. Bars represent the standard error of the mean.

Amygdala ROI parameter estimates from the group-level analysis showed that CS differentiation at CS onset was not consistent across acquisition, but instead varied as a function of learning block, as reflected in significant Cue × Block (*F* = 7.74, *p* = 0.01) and Cue × Time × Block (*F* = 6.12, *p* < 0.01) effects for the left amygdala (see Supplementary Table 1; Fig. 1a, b). In contrast, amygdala ROI parameter estimates began to discriminate between the offset of the non-reinforced CS + and the CS − over the course of acquisition, with the right amygdala showing a significant Cue × Time interaction effect (*F* = 2.49, *p* < 0.05) (see Supplementary Table 1; Fig. 1c, d). CS differentiation was clear from trials 5–8 and onward (Supplementary Table 2). During habituation, in the absence of learning, amygdala ROI parameter estimates for CS + and CS − did not differ significantly at cue onset or offset (all *p* > 0.05) (Supplementary Table 2).

### Insula ROI parameter estimates

**Fig. 2:**
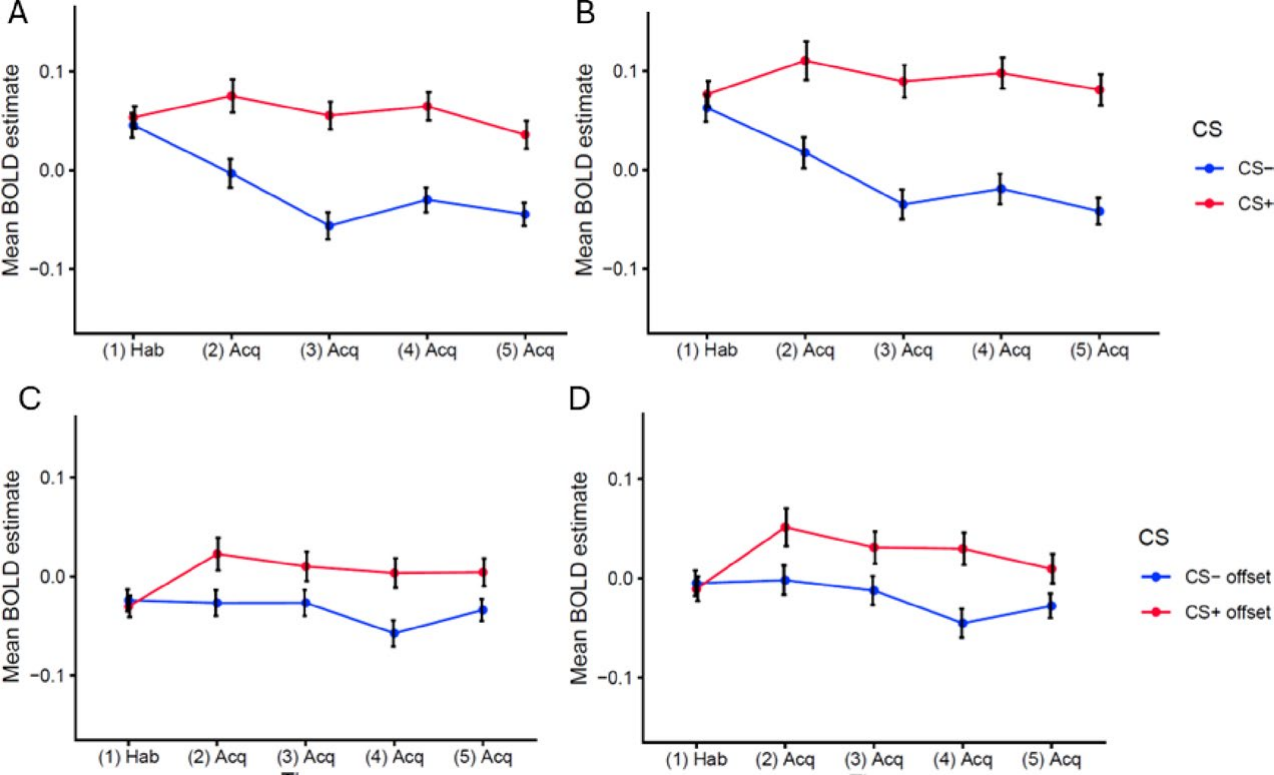
Insula ROI estimates to the onset of CS + and CS − presentations are shown in the (**A**) left and (**B**) right insula. Insula responses to the offset of CS + and CS− presentations are displayed for the (**C**) left and (**D**) right insula. Trials 1–4 are labeled as Hab, whereas trials 5–8, 9–12, 13–16, and 17–20 are labeled Acq 2–5. Bars represent the standard error of the mean.

Insula ROI parameter estimates from the group-level analysis showed differential responding to CS + versus CS − at CS onset that changed over the course of acquisition, as reflected in a Cue × Time interaction (*F* = 17.86, *p* < 0.001; Fig. 2). A similar learning-related Cue × Time effect was observed for CS offset responses (see Supplementary Table 1), indicating that the magnitude of CS differentiation at offset also varied across acquisition blocks.

## Whole brain analysis

**Fig. 3:**
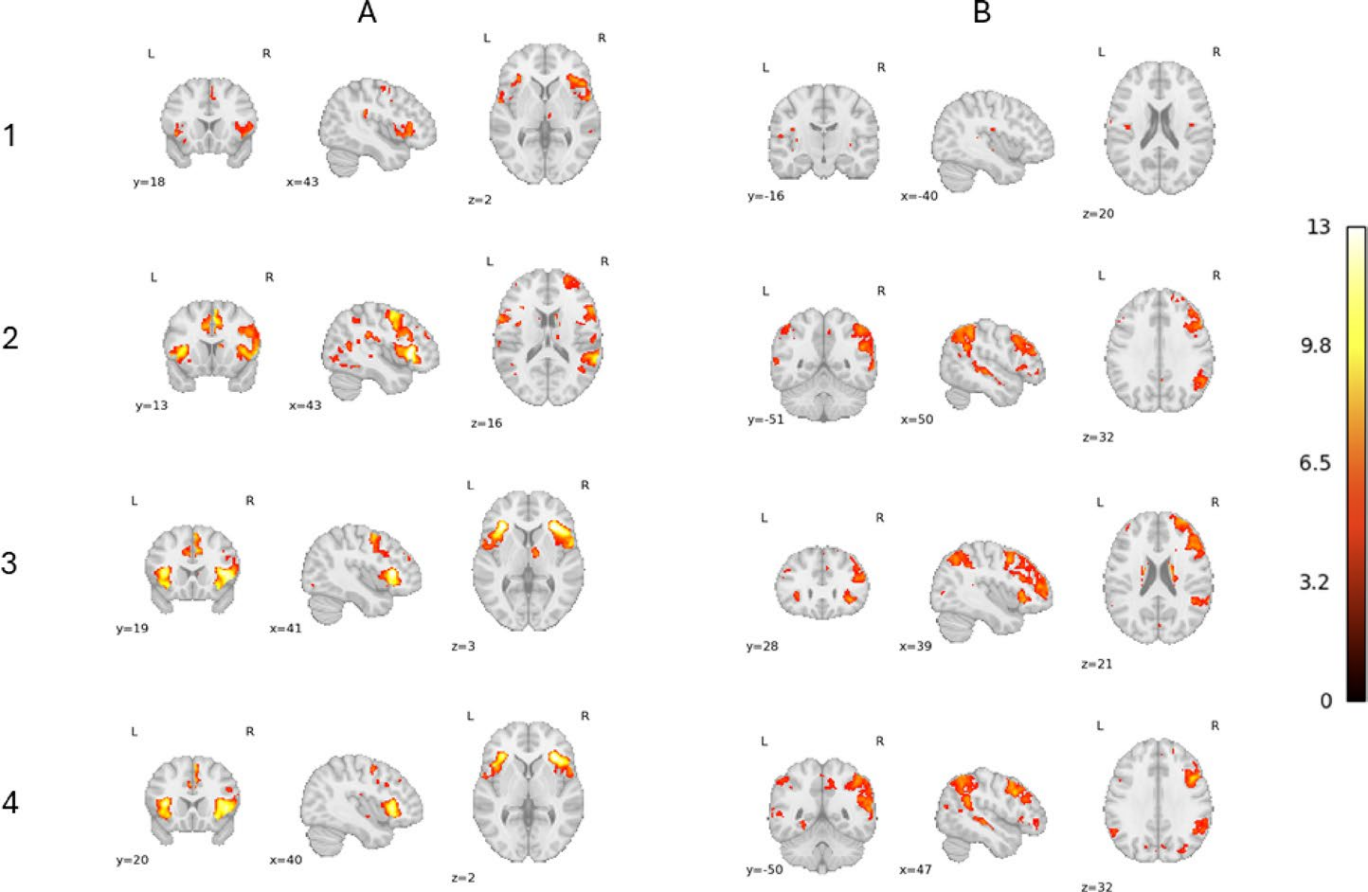
Results of voxel-wise whole brain analysis during acquisition of fear for trials (four blocks) 1) 5–8, 2) 9–12, 3) 13–16, 4) 17–20. The coronal, sagittal and axial slices show brain regions differentiating between (**A**) the onset of the conditioned stimulus associated with threat (CS +) compared to the control stimulus (CS −) and (**B**) the offset of CS + compared to the CS −. Bars represent t-scores, *p* < 0.05 FWE.

We contrasted onset and offset CS +  > CS − activation patterns across four acquisition blocks of trials (blocks of four trials each: trials 5–8, 9–12, 13–16, and 17–20). See Supplementary Tables 3,4 and Fig. 3 for full results. At CS onset, CS differentiation was initially observed (Trials 5–8) in the anterior insula and superior temporal gyrus. As learning advanced, activation patterns extended broadly to encompass regions such as the insula, thalamus, midbrain, temporal pole, inferior frontal gyrus, supramarginal gyrus, and middle frontal gyrus across both hemispheres.

**Fig. 4:**
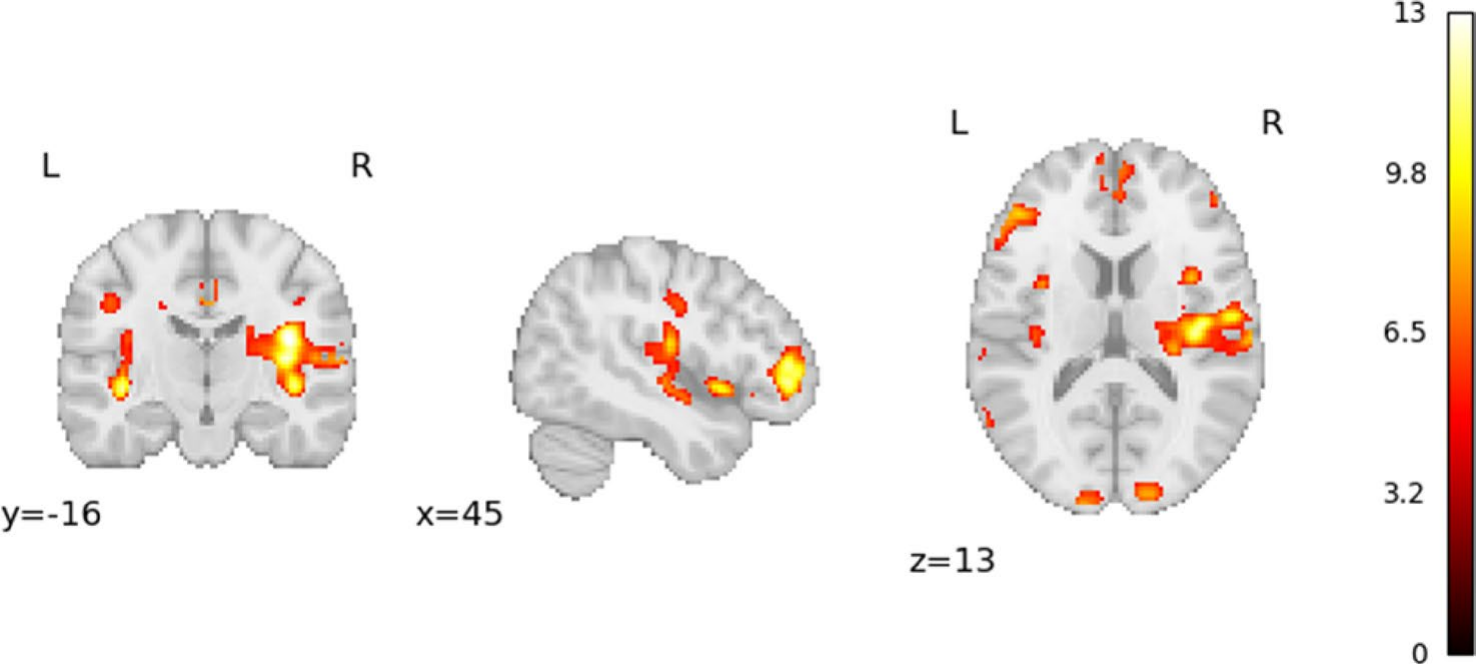
Voxel-wise whole brain analysis of responses to electric shock (US). Bars represent t-scores, *p* < 0.05 FWE.

In contrast, differential activation patterns to the offset of the CS + compared to the CS − recruited the posterior insula and the putamen. In subsequent trial blocks, activations associated with this contrast were observed to extended across extensive regions, including significant clusters within the right inferior frontal gyrus, middle frontal gyrus, angular gyrus, inferior parietal lobule, middle temporal gyrus, and caudate nucleus. By the later trial blocks, the offset response engaged a broader network of cortical and subcortical regions predominantly in the right hemisphere, which was the hemisphere contralateral to the arm to which the US was delivered. The whole-brain voxel-wise results for the US are depicted in Fig. 4 and Supplementary Table 5. Similar to the response pattern to the CS + offset when the US was omitted during the later trials (Fig. 3b), responses were lateralized to the side contralateral to the US delivery.

**Fig. 5:**
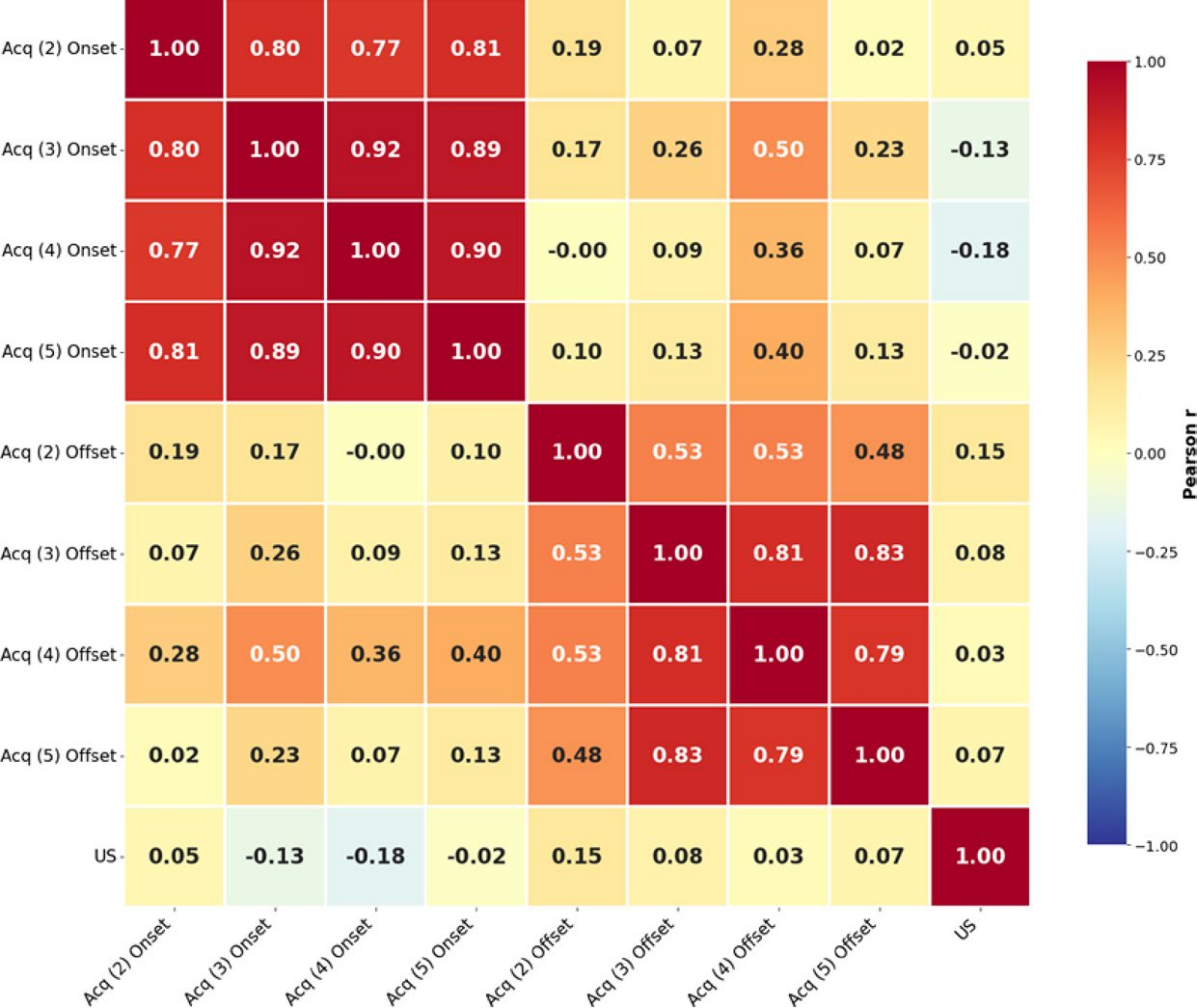
Correlation heat map illustrates the relationship between neural responses during the onset and offset phases of fear acquisition trials (four blocks) 5–8, 9–12, 13–16, 17–20 (Acq 2–5).

To further characterize similarity in whole-brain activation across learning, we generated a correlation heat map (Fig. 5) showing correlations of voxel-wise spatial activation patterns across the four acquisition blocks (trials 5–8, 9–12, 13–16, 17–20) for each event type (CS onset, CS offset on non-reinforced CS + trials, and US delivery). Here, “within onset” and “within offset” refer to correlations computed across acquisition blocks within the same event type (onset-with-onset; offset-with-offset). These within-event correlations were consistently high (*r* = 0.48–0.92), indicating that the spatial patterns were relatively stable across blocks for a given event type. In contrast, “between onset and offset phases” refers to correlations computed between different event types (onset versus offset), and we also examined correlations between each CS-related event type and the US. These between-event correlations were lower (*r* = −0.18–0.50). Overall, this descriptive pattern-similarity analysis suggests that whole-brain spatial activation patterns were more similar across blocks within the same event type than across different event types (onset, omission-related offset, and US delivery).

## Discussion

We investigated the dynamic activation patterns of the amygdala and insula to the onset and offset of conditioned stimuli over the course of fear conditioning in a large number of participants. We also contrasted responses to the offset and onset of conditioned stimuli across voxels in the whole brain. Findings revealed that both the amygdala and anterior insula exhibited changes in activity over the course of fear learning. The anterior insula started responding more to the onset and offset of the fear stimulus (CS +) than to the control stimulus (CS−) already during the first set of trials. The amygdala, on the other hand, responded only to CS offset, and at a later stage of learning than the anterior insula. Our whole brain analysis showed that differentiation at CS onset recruited larger brain networks as learning progressed. Activation patterns to the CS offset were predominantly lateralized to the hemisphere contralateral to US delivery. These findings show clear differences in the dynamic activation patterns related to the anticipation (onset) and reaction (offset) to learned threat. We also found that the neural response pattern to the US was clearly different to the response to the CS offset (US omission), implying that different processes drive these responses.

Results show that learning occurs not only to the onset of conditioned stimuli but also to their offset. This was evident because there was no differentiation between the offset of the CS + and CS − during the habituation phase, whereas differential responses increased across several brain areas over the course of acquisition when participants learned that the CS + predicted a shock to the left arm. Amygdala responses to CS + and CS − were indistinguishable at offset during habituation and the first four trials of the acquisition phase. However, as learning progressed, the amygdala responses began to show clear differentiation between the offset of the CS + and CS − , with significant discrimination emerging in later acquisition trials. In contrast, no consistent differentiation in amygdala responses was observed at cue onset throughout the acquisition phase. This pattern aligns with previous meta-analyses of neuroimaging studies of conditioned fear, which also report a lack of amygdala differentiation between CS types at onset ^7,11^. The results suggest that the amygdala’s involvement in threat processing is particularly sensitive to the expected moment of threat (CS + offset), rather than to the anticipation of threat at cue onset. Prior neuroimaging research has shown that amygdala activation rises as threats draw nearer^22^, possibly involving the centromedial region of the amygdala^23,24^. The amygdala is believed to help regulate defensive behaviors by supporting shifts from monitoring distant threats to initiating active defense when danger is close^25–27^. Furthermore, it may signal prediction errors related to the omission of an unconditioned stimulus, thereby updating the strength of CS-US associations and recalibrating threat expectations^18,28^.

The insula demonstrated a distinct pattern of activation as compared with the response pattern of the amygdala. As in the amygdala, insula responses during the habituation phase did not differ significantly between CS + and CS−. Yet, during acquisition, the insula exhibited robust and highly significant increases in response to the CS + compared with the CS −, especially at the onset, in contrast to the amygdala response pattern. Responses to the offset also clearly distinguished between the CS + and the CS − although the response difference was slightly smaller. These findings indicate that the insula is highly sensitive to the presence of conditioned threat cues, with significant activation at both the anticipation (onset) and the expected moment of threat (offset). This supports the insula’s established role in integrating emotional and interoceptive processes and in processing the anticipation and experience of aversive outcomes^7–9,11^.

We also compared whole-brain responses to the onsets and offsets of the CS + and CS −. This comparison highlights that, although both onset and offset activations evolved over the course of learning, offset responses showed a greater expansion in network involvement. Offset activation reflects the brain’s processing of threat omission and the updating of threat expectations, engaging higher-order associative and executive regions. Onset activation, meanwhile, is characterized by strong engagement of regions involved in anticipation and emotional processing, with less extensive recruitment of executive and associative cortices compared to offset. Thus, the neural response to the omission of threat (CS + termination) became more distributed and engaged a wider array of brain regions concentrated on the side contralateral to US delivery as fear learning progresses, whereas the response to the anticipation of threat (CS + onset) remains more focused in areas related to anticipation and emotional salience.

Another finding is that the neural response patterns were highly consistent within onset trials and within offset trials, indicating stable and distinct activation profiles for each phase. However, correlations between onset and offset phases were low, and the neural response pattern to the unconditioned stimulus (US) was not significantly correlated with either the onset or offset phases during fear learning. This suggests that the neural mechanisms underlying anticipation (onset), reaction (offset), and direct aversive experience (US) are functionally distinct and remain differentiated throughout the acquisition process.

This pattern is broadly consistent with influential psychological and neurobiological theories that emphasize prediction, outcome processing, and prediction-error signaling in threat learning^17–19^, as well as with prior neuroimaging work that has reported overlap between responses to predicted omissions and delivered aversive outcomes in some regions^14,16^. However, in the present data, whole-brain pattern similarity between CS-onset, CS-offset (non-reinforced CS +), and US responses was comparatively low. These results suggest that, at the level of large-scale BOLD activation patterns, responses time-locked to cue onset, omission-related cue offset, and US delivery can be partially dissociable. Given the correlational nature and temporal limitations of BOLD fMRI, these findings should not be interpreted as evidence for uniquely separable “processes,” but rather as consistent with differential engagement across event types during acquisition.

### Limitations

Several limitations should be acknowledged. The present sample consisted of adult twins recruited through the Swedish Twin Registry, reflecting the sampling frame of the parent project. The current manuscript does not apply twin modeling (e.g., heritability or within-pair similarity) and should therefore be interpreted primarily as a characterization of neural responses in a relatively large adult sample. Although there is no clear reason to expect that twins to respond differently from non-twins in this type of conditioning task, replication in more diverse samples would strengthen generalizability. The use of fMRI, while powerful for spatial localization, has limited temporal resolution compared to electrophysiological methods, which constrains inferences about rapid neural dynamics. Additionally, the study focused on habituation and acquisition; extinction was not examined and may yield different patterns of responding at CS onset and CS offset.

### Conclusion

In conclusion, the onset and offset of conditioned cues were associated with different patterns of BOLD activation over learning. Amygdala ROI estimates showed the most consistent CS differentiation at CS + offset on non-reinforced CS + trials (i.e., when the US was expected but omitted), whereas insula ROI estimates differentiated CS + from CS − at both CS onset and CS offset. Whole-brain analyses further indicated that CS-onset and CS-offset contrasts engaged partly different large-scale networks across acquisition blocks. Together, these results are consistent with differential engagement of amygdala and insula across event types during threat learning, while highlighting the need for caution when drawing mechanistic inferences from BOLD fMRI alone.

## Materials and methods

### Subjects

Twins aged 20–60 were recruited from the Swedish Twin Registry (STR). Same-sex twin pairs with known zygosity were selected if eligible for MRI and screened for substance abuse, psychological treatment, or medications affecting emotion or cognition. After initial screening, 305 participants underwent fMRI scanning. Participants were excluded due to excessive head motion (> 50% frames above 0.5 mm displacement) *n* = 16 and missing data *n* = 3. The final sample included 286 twins: 69 identical pairs (39 female, 30 male; mean age 31) and 74 fraternal pairs (43 female, 31 male; mean age 31). All participants gave written informed consent per Uppsala Ethical Review Board guidelines and received SEK 1000 (~ 100 USD) compensation. The study was conducted in accordance with the guidelines and regulations outlined by the Uppsala Ethical Review Board (Dnr 2014-01,160).

### Brain imaging

Imaging data were collected with a 3.0 T Discovery MR750 scanner (GE Healthcare) and an 8-channel head coil. Foam wedges, earplugs, and headphones were used to minimize head motion and noise. T1-weighted structural images were acquired with whole head coverage (TR = 6400 ms, TE = 3 ms, 6.04 min, flip angle 11°). Functional images used gradient EPI (TR = 2390 ms, TE = 28 ms, flip angle = 80°, 47 volumes, 3.0 mm slice thickness, axial, A/P phase-encoding). Slices were acquired interleaved and ascending. Higher order shimming and 5 dummy scans were acquired before data acquisition.

### Stimuli and contexts

Visual stimuli were displayed on a flat MR scanner screen via an Epson EX5260 projector. Stimulus presentation was run on a custom Unity 5.2.3 build and interfaced with BIOPAC for electrical stimuli through a parallel port using custom .NET serial communication software.

### fMRI paradigm design

Participants engaged in a delayed threat-conditioning protocol during functional magnetic resonance imaging (fMRI) sessions. Two humanoid virtual characters (see Supplementary Fig. 1) served as conditioned cues (CS): one CS was designated CS + (threat cue) and the other CS − (safety cue); CS assignment to character identity was counterbalanced across participants. Each trial consisted of cue presentation (6 s) followed by a jittered inter-trial interval (8–12 s). The CS + was partially reinforced: on 50% of CS + trials, a brief electrical shock (US) was delivered at the end of the cue (co-terminating with CS + offset); on the remaining CS + trials, no shock was delivered (non-reinforced CS + trials), enabling analysis of responses time-locked to CS + offset when the US was expected but omitted. The CS − was never paired with shock. The task began with a habituation phase comprising four non-reinforced presentations of each cue (no US delivered), followed by an acquisition phase comprising 16 presentations of each cue (32 acquisition trials total). Trial order during habituation and acquisition was pseudo-randomized and counterbalanced across participants, with constraints to avoid long runs of the same cue and to distribute reinforced CS + trials across acquisition. The full task lasted 9 min 47 s; all phases were included in the modeling framework described below.

The electrical shocks were delivered to the distal part of the participant’s left volar forearm (adjacent to the wrist) via radio-translucent disposable dry electrodes (EL509, BIOPAC Systems, Goleta, CA). Shock delivery was controlled using the STM100C module connected to the STM200 constant current stimulator (BIOPAC Systems), using a unipolar pulse with a fixed duration of 67 Hz. The voltage level was individually calibrated before the experimental task using an ascending staircase procedure until shocks were rated as “aversive”^29^. After finding the voltage level that participants rated as aversive, this parameter was kept constant throughout the experiment. The determined average electrical voltage was *M* = 31 V, *SD* = 7, and range = (15, 55).

### Analysis of fMRI imaging data

fMRI data were analyzed using ENIGMA HALFpipe^30^. Preprocessing involved slice timing correction, realignment, co-registration to T1 images, normalization to MNI152NLin6Asym space and ICA-AROMA filtering for artifact removal.

First-level analysis used an event-related general linear model (GLM) implemented in SPM12 (Wellcome Centre for Human Imaging). Regressors of interest were defined to capture responses at distinct moments within the conditioning trial: (1) CS onset responses, modeled as a 6 s event beginning at CS onset (separately for CS + and CS −); (2) CS offset responses, modeled as a 6 s event beginning at cue offset, defined for non-reinforced CS + trials (i.e., CS + offset when the US was expected but omitted) and for CS − offset; and (3) US responses, modeled as a 1 s event beginning at US onset (reinforced CS + trials only). All task regressors were convolved with the canonical hemodynamic response function. Nuisance regressors included standard motion parameters and ICA-AROMA components as implemented in HALFpipe.

**Fig. 6:**
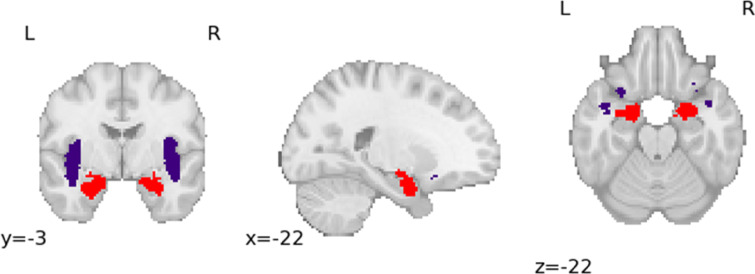
This figure illustrates the anatomical regions of interest that were applied to the fMRI data to extract neural responses during the fear conditioning task. The colored overlays highlight the bilateral amygdala (red) and insula (purple).

To examine learning-related dynamics, habituation trials (trials 1–4 per cue) were modeled separately from acquisition. Acquisition was indexed using blocks of four trials (trials 5–8, 9–12, 13–16, 17–20), which were included as a within-subject Time/Block factor in group-level analyses of ROI estimates and contrasts. Whole-brain voxel-wise analyses were conducted for block-wise contrasts, and statistical significance was assessed using family-wise error (FWE) correction at *p* < 0.05. Regions of interest (ROIs) were defined anatomically for bilateral amygdala and insula (Fig. 6). Anatomical labeling of significantly activated brain regions were performed using the Jülich brain atlas v29^31^. For each participant and event regressor, parameter estimates were averaged within each ROI, using the spm_read_vols function in SPM12. Left and right hemispheres were treated as separate a priori ROIs and are reported separately throughout (laterality was not a primary focus of this report).

### Statistical analyses

Group-level analyses tested whether ROI-averaged parameter estimates differed as a function of Cue (CS + vs CS −), Event (onset vs offset), and learning progression (Habituation vs Acquisition blocks), with primary inferences focused on Cue effects and their interactions with Time/Block and/or Event. Planned simple-effects follow-ups were conducted only when supported by significant omnibus interaction terms (e.g., Cue × Time/Block), and follow-up tests were corrected for multiple comparisons. Because participants were twins, inferential models accounted for non-independence within twin pairs by using cluster-robust inference at the family (twin-pair) level.

## Supplementary Information

Below is the link to the electronic supplementary material.

Supplementary Material 1

## Data Availability

All data generated during the current study can be made available upon reasonable request from the corresponding author.
