# Supplementary material for: Dynamic changes in amygdala and insula responses to the onset and offset of conditioned stimuli during threat learning
Source: Sci Rep. 2026 Jul 17;16:22512. doi: 10.1038/s41598-026-62910-8 (PMC13379584; doi:10.1038/s41598-026-62910-8)
Supplement: Supplementary file 1 — Supplementary Material 1 [file 41598_2026_62910_MOESM1_ESM.docx]

**Supplementary materials**

**Supplementary Table 1.** Results of the ANOVA analyses conducted on amygdala and insula activation during the fear conditioning task. The table details the effects of different experimental variables, conditioned stimulus (Cue), time, block, and their interactions, on neural responses in the left and right amygdala and insula. For each brain region, the table lists the degrees of freedom (df), F-values, p-values, and Bonferroni-corrected significance levels for the main effects and interactions. Statistically significant effects are marked with an asterisk.

| Brain region | Variable | df | F | p | p < 0.05 (bonferroni) |
| --- | --- | --- | --- | --- | --- |
| Amygdala L | Cue | 285 | 6.06 | 0.01 | * |
|  | Time | 285 | 1.41 | 0.24 |  |
|  | Block | 285 | 0.19 | 0.66 |  |
|  | Cue x Time | 285 | 1.03 | 0.39 |  |
|  | Cue x Block | 285 | 7.74 | 0.01 | * |
|  | Time x Block | 285 | 15.39 | 0.00 | * |
|  | Cue x Time x Block | 285 | 6.12 | 0.00 | * |
|  |  |  |  |  |  |
| Amygdala R | Cue | 285 | 19.36 | 0.00 | * |
|  | Time | 285 | 2.50 | 0.05 |  |
|  | Block | 285 | 0.26 | 0.61 |  |
|  | Cue x Time | 285 | 2.49 | 0.05 | * |
|  | Cue x Block | 285 | 16.41 | 0.00 | * |
|  | Time x Block | 285 | 10.49 | 0.00 | * |
|  | Cue x Time x Block | 285 | 5.94 | 0.00 | * |
|  |  |  |  |  |  |
| Insula R | Cue | 285 | 162.65 | 0.00 | * |
|  | Time | 285 | 1.65 | 0.17 |  |
|  | Block | 285 | 48.99 | 0.00 | * |
|  | Cue x Time | 285 | 16.17 | 0.00 | * |
|  | Cue x Block | 285 | 23.25 | 0.00 | * |
|  | Time x Block | 285 | 12.73 | 0.00 | * |
|  | Cue x Time x Block | 285 | 2.53 | 0.04 | * |
|  |  |  |  |  |  |
| Insula L | Cue | 285 | 218.62 | 0.00 | * |
|  | Time | 285 | 2.34 | 0.07 |  |
|  | Block | 285 | 62.56 | 0.00 | * |
|  | Cue x Time | 285 | 17.86 | 0.00 | * |
|  | Cue x Block | 285 | 38.52 | 0.00 | * |
|  | Time x Block | 285 | 7.34 | 0.00 | * |
|  | Cue x Time x Block | 285 | 3.37 | 0.01 | * |

**Supplementary Table 2**. Presents the results of post-hoc t-tests comparing amygdala and insula activation at both the onset and offset of the conditioned stimulus (CS+) versus the control stimulus (CS-) across different phases and trials of fear conditioning. The table details the statistical significance, direction, and timing of neural discrimination between cues. The trials from the fear conditioning task were grouped into blocks to facilitate statistical comparisons of neural activation across different learning phases. The initial four trials for each cue constituted the "habituation" phase, during which no shocks were delivered and no conditioned learning was expected. Subsequent trials were divided into blocks of four, representing progressive stages of the "acquisition" phase (e.g., trials 5–8, 9–12, 13–16, and 17–20).

| Brain region | t-test | df | p < 0.05 (bonferroni) | Block | Time |
| --- | --- | --- | --- | --- | --- |
| Amygdala L | 0.61 | 285 | ns | (1) Hab | onset |
| Amygdala R | 0.95 | 285 | ns | (1) Hab | onset |
| Insula L | 1.03 | 285 | ns | (1) Hab | onset |
| Insula R | 1.71 | 285 | ns | (1) Hab | onset |
| Amygdala L | -0.47 | 285 | ns | (1) Hab | offset |
| Amygdala R | 0.11 | 285 | ns | (1) Hab | offset |
| Insula L | -0.91 | 285 | ns | (1) Hab | offset |
| Insula R | -0.77 | 285 | ns | (1) Hab | offset |
| Amygdala L | 2.12 | 285 | * | (2) Acq | onset |
| Amygdala R | 1.75 | 285 | ns | (2) Acq | onset |
| Insula L | 7.08 | 285 | **** | (2) Acq | onset |
| Insula R | 7.58 | 285 | **** | (2) Acq | onset |
| Amygdala L | 0.78 | 285 | ns | (3) Acq | onset |
| Amygdala R | 1.74 | 285 | ns | (3) Acq | onset |
| Insula L | 9.58 | 285 | **** | (3) Acq | onset |
| Insula R | 9.73 | 285 | **** | (3) Acq | onset |
| Amygdala L | -1.87 | 285 | ns | (4) Acq | onset |
| Amygdala R | -1.94 | 285 | ns | (4) Acq | onset |
| Insula L | 7.51 | 285 | **** | (4) Acq | onset |
| Insula R | 8.71 | 285 | **** | (4) Acq | onset |
| Amygdala L | -2.06 | 285 | * | (5) Acq | onset |
| Amygdala R | -2.02 | 285 | * | (5) Acq | onset |
| Insula L | 6.01 | 285 | **** | (5) Acq | onset |
| Insula R | 8.07 | 285 | **** | (5) Acq | onset |
| Amygdala L | 0.25 | 285 | ns | (2) Acq | offset |
| Amygdala R | 1.44 | 285 | ns | (2) Acq | offset |
| Insula L | 4.34 | 285 | **** | (2) Acq | offset |
| Insula R | 4.49 | 285 | **** | (2) Acq | offset |
| Amygdala L | 2.91 | 285 | ** | (3) Acq | offset |
| Amygdala R | 4.68 | 285 | **** | (3) Acq | offset |
| Insula L | 3.28 | 285 | *** | (3) Acq | offset |
| Insula R | 3.61 | 285 | *** | (3) Acq | offset |
| Amygdala L | 2.91 | 285 | ** | (4) Acq | offset |
| Amygdala R | 4.04 | 285 | **** | (4) Acq | offset |
| Insula L | 5.09 | 285 | **** | (4) Acq | offset |
| Insula R | 6.00 | 285 | **** | (4) Acq | offset |
| Amygdala L | 3.24 | 285 | *** | (5) Acq | offset |
| Amygdala R | 3.62 | 285 | *** | (5) Acq | offset |
| Insula L | 3.25 | 285 | *** | (5) Acq | offset |
| Insula R | 2.73 | 285 | ** | (5) Acq | offset |

**Supplementary Table 3**. Detailed overview of the evolving spatial distribution and intensity of brain activation across the four blocks of trials (e.g., trials 5–8, 9–12, 13–16, and 17–20) during the onset of the conditioned stimulus (CS+) over the control stimulus (CS-). The table lists the major cortical and subcortical regions that show significant activation for each trial, including cluster sizes and peak statistical values (p < 0.05 FWE).

|  | |  |  | MNI Coordinates | | |
| --- | --- | --- | --- | --- | --- | --- |
|  | Brain region | Cluster size | t-values | x | y | z |
| **Block 1** | R Rolandic Operculum | 641 | 9.15 | 54 | -31 | 26 |
|  | Location not in atlas | 1048 | 8.71 | 36 | 28 | 6 |
|  | R Temporal Pole | 1048 | 8.38 | 58 | 2 | 6 |
|  | R Insula Lobe | 1048 | 5.58 | 36 | 8 | 10 |
|  | L Superior Temporal Gyrus | 86 | 8.23 | -41 | -7 | -5 |
|  | L IFG (p. Orbitalis) | 434 | 7.72 | -43 | 18 | -3 |
|  | L Insula Lobe | 434 | 6.65 | -31 | 22 | 14 |
|  | Location not in atlas | 35 | 7.65 | -13 | -23 | 40 |
|  | Location not in atlas | 128 | 7.56 | 36 | -5 | -3 |
|  | L Superior Temporal Gyrus | 109 | 7.37 | -59 | -1 | 4 |
|  | Location not in atlas | 89 | 7.04 | 12 | -21 | 42 |
|  | R Postcentral Gyrus | 157 | 6.96 | 30 | -43 | 66 |
|  | R Posterior-Medial Frontal | 35 | 6.88 | 10 | -7 | 68 |
|  | R MCC | 212 | 6.85 | 4 | 14 | 36 |
|  | R Posterior-Medial Frontal | 212 | 6.66 | 6 | 6 | 56 |
|  | R Superior Medial Gyrus | 45 | 6.39 | 6 | 32 | 52 |
|  | L SupraMarginal Gyrus | 104 | 6.31 | -65 | -47 | 30 |
|  | R Thalamus | 16 | 6.31 | 6 | -21 | 6 |
|  | R Precuneus | 27 | 6.18 | 6 | -49 | 60 |
|  | R Superior Temporal Gyrus | 44 | 6.17 | 58 | -41 | 16 |
|  | R Precentral Gyrus | 79 | 6.12 | 52 | 2 | 48 |
|  | L ACC | 9 | 5.91 | -5 | 22 | 32 |
|  | L MCC | 7 | 5.84 | -5 | 6 | 42 |
|  | R ACC | 30 | 5.71 | 10 | 34 | 32 |
|  | L Insula Lobe | 9 | 5.60 | -37 | -1 | 14 |
|  | R Middle Temporal Gyrus | 6 | 5.48 | 60 | -39 | 4 |
|  | R Insula Lobe | 11 | 5.42 | 40 | -17 | 22 |
|  | R MCC | 6 | 5.25 | 6 | -31 | 52 |
|  | L Insula Lobe | 7 | 5.24 | -45 | 4 | 2 |
|  |  |  |  |  |  |  |
| **Block 2** | Location not in atlas | 3909 | 15.15 | 34 | 28 | 4 |
|  | R Precentral Gyrus | 3909 | 11.79 | 48 | -1 | 50 |
|  | R Rolandic Operculum | 3909 | 10.84 | 58 | 4 | 8 |
|  | L IFG (p. Orbitalis) | 2123 | 14.47 | -31 | 28 | -1 |
|  | L Temporal Pole | 2123 | 10.34 | -59 | 4 | 4 |
|  | L Insula Lobe | 2123 | 7.33 | -37 | -1 | 12 |
|  | R SupraMarginal Gyrus | 2874 | 11.12 | 62 | -45 | 32 |
|  | R Middle Temporal Gyrus | 2874 | 9.38 | 56 | -41 | 12 |
|  | R Rolandic Operculum | 2874 | 8.59 | 64 | -23 | 22 |
|  | R MCC | 2336 | 10.96 | 8 | 12 | 44 |
|  | R Superior Medial Gyrus | 2336 | 10.47 | 4 | 34 | 52 |
|  | R Posterior-Medial Frontal | 2336 | 9.70 | 12 | -5 | 66 |
|  | Location not in atlas | 279 | 10.49 | 6 | -23 | -1 |
|  | L MCC | 784 | 10.40 | -5 | 8 | 40 |
|  | L Posterior-Medial Frontal | 784 | 9.27 | -7 | -9 | 72 |
|  | L ACC | 784 | 7.36 | -5 | 26 | 30 |
|  | R Postcentral Gyrus | 428 | 10.13 | 22 | -47 | 72 |
|  | R Postcentral Gyrus | 428 | 6.34 | 36 | -37 | 58 |
|  | L SupraMarginal Gyrus | 1025 | 9.04 | -61 | -47 | 32 |
|  | L SupraMarginal Gyrus | 1025 | 8.66 | -65 | -29 | 22 |
|  | L Precentral Gyrus | 295 | 8.92 | -45 | -7 | 50 |
|  | R Middle Frontal Gyrus | 831 | 8.85 | 36 | 48 | 26 |
|  | Location not in atlas | 178 | 8.48 | -13 | -25 | 42 |
|  | R Caudate Nucleus | 33 | 7.85 | 10 | 14 | 10 |
|  | Location not in atlas | 16 | 7.70 | -5 | -23 | -1 |
|  | Location not in atlas | 38 | 7.55 | -7 | -33 | -15 |
|  | R Fusiform Gyrus | 113 | 7.38 | 38 | -61 | -15 |
|  | R Inferior Occipital Gyrus | 37 | 7.12 | 26 | -95 | -5 |
|  | L Middle Frontal Gyrus | 210 | 6.92 | -41 | 40 | 32 |
|  | L Middle Frontal Gyrus | 210 | 5.76 | -39 | 52 | 12 |
|  | Location not in atlas | 24 | 6.76 | -11 | -5 | 8 |
|  | L Inferior Occipital Gyrus | 57 | 6.72 | -41 | -85 | -5 |
|  | L Middle Temporal Gyrus | 174 | 6.69 | -49 | -63 | 12 |
|  | R Cuneus | 112 | 6.68 | 14 | -75 | 40 |
|  | R Inferior Occipital Gyrus | 141 | 6.44 | 42 | -81 | -9 |
|  | L Superior Medial Gyrus | 25 | 6.31 | -9 | 38 | 36 |
|  | Location not in atlas | 8 | 6.09 | 30 | 60 | -7 |
|  | R Superior Frontal Gyrus | 9 | 6.07 | 20 | 8 | 62 |
|  | Location not in atlas | 13 | 6.05 | -43 | -51 | 38 |
|  | Location not in atlas | 8 | 6.00 | 16 | -3 | 18 |
|  | L Postcentral Gyrus | 51 | 5.98 | -23 | -47 | 72 |
|  | Location not in atlas | 31 | 5.91 | -3 | -23 | 32 |
|  | L Precentral Gyrus | 27 | 5.84 | -51 | -3 | 38 |
|  |  |  |  |  |  |  |
| **Block 3** | R Insula Lobe | 2342 | 15.40 | 34 | 26 | 6 |
|  | R Temporal Pole | 2342 | 9.43 | 56 | 6 | 4 |
|  | R Precentral Gyrus | 2342 | 9.40 | 46 | -1 | 52 |
|  | L IFG (p. Orbitalis) | 1397 | 14.87 | -33 | 28 | -1 |
|  | L IFG (p. Opercularis) | 1397 | 8.20 | -55 | 8 | 8 |
|  | L Insula Lobe | 1397 | 5.94 | -37 | -1 | 12 |
|  | Location not in atlas | 241 | 10.79 | 6 | -31 | -9 |
|  | Location not in atlas | 241 | 7.58 | 12 | -9 | 6 |
|  | R Posterior-Medial Frontal | 1277 | 10.68 | 6 | 10 | 52 |
|  | R Superior Medial Gyrus | 1277 | 9.85 | 6 | 38 | 46 |
|  | R Posterior-Medial Frontal | 1277 | 6.90 | 14 | -5 | 66 |
|  | R Rolandic Operculum | 1139 | 8.91 | 50 | -29 | 28 |
|  | R Superior Temporal Gyrus | 1139 | 8.26 | 56 | -45 | 16 |
|  | R SupraMarginal Gyrus | 1139 | 7.24 | 56 | -49 | 36 |
|  | L MCC | 372 | 8.83 | -5 | 8 | 46 |
|  | L ACC | 372 | 6.71 | -7 | 26 | 30 |
|  | L Posterior-Medial Frontal | 76 | 8.70 | -11 | -7 | 70 |
|  | Location not in atlas | 39 | 8.54 | -5 | -31 | -7 |
|  | L SupraMarginal Gyrus | 583 | 8.41 | -59 | -49 | 30 |
|  | L SupraMarginal Gyrus | 583 | 7.17 | -65 | -29 | 26 |
|  | R Caudate Nucleus | 19 | 8.31 | 10 | 14 | 10 |
|  | R Postcentral Gyrus | 81 | 7.94 | 22 | -49 | 70 |
|  | R Middle Temporal Gyrus | 29 | 7.85 | 52 | -29 | -5 |
|  | R Middle Frontal Gyrus | 447 | 7.75 | 32 | 52 | 22 |
|  | R Middle Frontal Gyrus | 447 | 6.63 | 38 | 38 | 36 |
|  | L Precentral Gyrus | 140 | 7.73 | -47 | -5 | 50 |
|  | Location not in atlas | 50 | 7.69 | -13 | -25 | 40 |
|  | Location not in atlas | 20 | 6.89 | -5 | -15 | -3 |
|  | L Superior Temporal Gyrus | 21 | 6.77 | -41 | -9 | -5 |
|  | Location not in atlas | 39 | 6.35 | 14 | -27 | 42 |
|  | Location not in atlas | 10 | 6.12 | 36 | -7 | -7 |
|  | R Precuneus | 30 | 6.01 | 16 | -67 | 40 |
|  | L Inferior Occipital Gyrus | 9 | 5.74 | -33 | -95 | -3 |
|  | R MCC | 7 | 5.62 | 2 | -21 | 42 |
|  | Location not in atlas | 8 | 5.58 | -7 | -21 | 28 |
|  | Location not in atlas | 7 | 5.54 | 4 | -23 | 30 |
|  | L Middle Frontal Gyrus | 8 | 5.45 | -37 | 46 | 24 |
|  | L Middle Temporal Gyrus | 5 | 5.29 | -57 | -51 | 14 |
|  |  |  |  |  |  |  |
| **Block 4** | R IFG (p. Orbitalis) | 1523 | 14.04 | 34 | 30 | -3 |
|  | R Temporal Pole | 1523 | 7.73 | 58 | 4 | 6 |
|  | R Insula Lobe | 1523 | 6.20 | 38 | 4 | 8 |
|  | L IFG (p. Triangularis) | 1056 | 12.88 | -35 | 24 | 8 |
|  | L Temporal Pole | 1056 | 6.98 | -57 | 6 | 4 |
|  | R Superior Medial Gyrus | 898 | 9.07 | 4 | 24 | 54 |
|  | R ACC | 898 | 7.70 | 4 | 28 | 34 |
|  | R MCC | 898 | 7.30 | 4 | -1 | 44 |
|  | L Superior Temporal Gyrus | 554 | 8.04 | -61 | -35 | 28 |
|  | R Superior Frontal Gyrus | 241 | 7.99 | 26 | 54 | 22 |
|  | R SupraMarginal Gyrus | 915 | 7.89 | 64 | -45 | 36 |
|  | R Middle Temporal Gyrus | 915 | 7.11 | 58 | -47 | 10 |
|  | R Superior Temporal Gyrus | 915 | 6.47 | 60 | -29 | 20 |
|  | R Posterior-Medial Frontal | 44 | 7.73 | 14 | -5 | 66 |
|  | R Precentral Gyrus | 218 | 7.50 | 42 | -3 | 52 |
|  | L ACC | 102 | 7.44 | -5 | 22 | 32 |
|  | R Postcentral Gyrus | 71 | 7.42 | 22 | -47 | 68 |
|  | R Middle Temporal Gyrus | 46 | 7.13 | 52 | -27 | -5 |
|  | Location not in atlas | 59 | 6.91 | 38 | -19 | -3 |
|  | Location not in atlas | 59 | 5.54 | 36 | 2 | -7 |
|  | Location not in atlas | 13 | 6.67 | 6 | -29 | -9 |
|  | R Caudate Nucleus | 9 | 6.67 | 10 | 14 | 10 |
|  | Location not in atlas | 11 | 6.54 | 6 | -21 | -1 |
|  | R IFG (p. Triangularis) | 65 | 6.50 | 46 | 20 | 26 |
|  | L Precentral Gyrus | 40 | 6.47 | -43 | -7 | 52 |
|  | L Insula Lobe | 11 | 6.46 | -37 | -7 | -5 |
|  | Location not in atlas | 10 | 5.98 | 10 | -7 | 8 |
|  | R Insula Lobe | 11 | 5.84 | 40 | -19 | 20 |
|  | L Posterior-Medial Frontal | 12 | 5.77 | -11 | -7 | 72 |
|  | Location not in atlas | 16 | 5.70 | -13 | -23 | 40 |

**Supplementary Table 4**. Detailed overview of the evolving spatial distribution and intensity of brain activation across four blocks (e.g., trials 5–8, 9–12, 13–16, and 17–20) during the offset of the conditioned stimulus (CS+) over the control stimulus (CS-). The table lists the major cortical and subcortical regions that show significant activation for each trial, including cluster sizes and peak statistical values (p < 0.05 FWE)

|  | |  |  | MNI Coordinates | | |
| --- | --- | --- | --- | --- | --- | --- |
|  | Brain region | Cluster size | t-values | x | y | z |
| **Block 1** | R Putamen | 15 | 6.65 | 34 | -7 | -1 |
|  | L Insula Lobe | 10 | 6.20 | -35 | -25 | 14 |
|  | L Insula Lobe | 11 | 6.17 | -37 | -5 | -1 |
|  | L Rolandic Operculum | 18 | 5.88 | -41 | -17 | 22 |
|  | L Superior Temporal Gyrus | 16 | 5.88 | -55 | -19 | 12 |
|  | R Insula Lobe | 17 | 5.84 | 44 | -13 | 18 |
|  | R Putamen | 5 | 5.72 | 34 | -15 | -1 |
|  | R Insula Lobe | 5 | 5.64 | 40 | -3 | -7 |
|  | R SupraMarginal Gyrus | 7 | 5.57 | 56 | -37 | 54 |
|  | L Heschls Gyrus | 5 | 5.56 | -49 | -15 | 16 |
|  | L Precentral Gyrus | 5 | 5.53 | -31 | -27 | 56 |
|  | R Middle Frontal Gyrus | 8 | 5.51 | 32 | 18 | 56 |
|  | Location not in atlas | 5 | 5.47 | 42 | -11 | -9 |
|  | L Precentral Gyrus | 13 | 5.47 | -57 | -7 | 28 |
|  | R Precentral Gyrus | 9 | 5.38 | 60 | -3 | 32 |
|  |  |  |  |  |  |  |
| **Block 2** | R IFG (p. Triangularis) | 2048 | 8.88 | 56 | 18 | 32 |
|  | R Middle Frontal Gyrus | 2048 | 7.53 | 44 | 40 | 30 |
|  | R Middle Frontal Gyrus | 2048 | 7.44 | 32 | 20 | 58 |
|  | R Angular Gyrus | 2474 | 8.44 | 56 | -55 | 30 |
|  | R Inferior Parietal Lobule | 2474 | 8.18 | 46 | -55 | 56 |
|  | R Middle Temporal Gyrus | 2474 | 8.00 | 62 | -45 | 2 |
|  | R Caudate Nucleus | 63 | 7.46 | 14 | 6 | 18 |
|  | R Cerebelum (VI) | 390 | 7.43 | 28 | -83 | -13 |
|  | R Middle Occipital Gyrus | 390 | 6.66 | 32 | -91 | 6 |
|  | R Linual Gyrus | 390 | 5.30 | 26 | -65 | -3 |
|  | R Caudate Nucleus | 16 | 7.28 | 18 | -11 | 24 |
|  | R IFG (p. Orbitalis) | 243 | 7.22 | 42 | 24 | -7 |
|  | R Precuneus | 305 | 7.19 | 2 | -63 | 46 |
|  | L Caudate Nucleus | 5 | 7.18 | -17 | -5 | 22 |
|  | L Middle Temporal Gyrus | 168 | 7.15 | -61 | -41 | -1 |
|  | L Middle Temporal Gyrus | 168 | 5.26 | -61 | -57 | 18 |
|  | L IFG (p. Orbitalis) | 109 | 6.80 | -45 | 16 | -5 |
|  | R Middle Temporal Gyrus | 46 | 6.73 | 52 | -11 | -15 |
|  | L Inferior Parietal Lobule | 289 | 6.71 | -53 | -55 | 52 |
|  | L Postcentral Gyrus | 289 | 5.33 | -45 | -29 | 48 |
|  | L Fusiform Gyrus | 22 | 6.48 | -23 | -81 | -7 |
|  | L IFG (p. Opercularis) | 26 | 6.35 | -47 | 12 | 28 |
|  | R ACC | 60 | 6.23 | 8 | 48 | 14 |
|  | L Middle Frontal Gyrus | 54 | 6.17 | -31 | 12 | 58 |
|  | R Superior Frontal Gyrus | 22 | 6.15 | 22 | 32 | 38 |
|  | R Precentral Gyrus | 7 | 6.10 | 60 | 4 | 30 |
|  | R Linual Gyrus | 55 | 6.05 | 4 | -87 | -1 |
|  | R Superior Medial Gyrus | 13 | 6.01 | 6 | 44 | 52 |
|  | L IFG (p. Triangularis) | 12 | 5.90 | -51 | 28 | 30 |
|  | L Putamen | 5 | 5.86 | -29 | -25 | 8 |
|  | L Fusiform Gyrus | 15 | 5.76 | -29 | -65 | -7 |
|  | R Middle Frontal Gyrus | 14 | 5.74 | 24 | 48 | 36 |
|  | Location not in atlas | 16 | 5.72 | -49 | 44 | -7 |
|  | L Middle Frontal Gyrus | 17 | 5.72 | -41 | 20 | 50 |
|  | R MCC | 6 | 5.64 | 4 | 24 | 42 |
|  | L Precentral Gyrus | 14 | 5.56 | -43 | 6 | 52 |
|  | R Calcarine Gyrus | 14 | 5.55 | 16 | -91 | 14 |
|  | L Precentral Gyrus | 19 | 5.54 | -53 | 6 | 44 |
|  | L Middle Frontal Gyrus | 13 | 5.51 | -43 | 24 | 42 |
|  | L Inferior Occipital Gyrus | 16 | 5.51 | -33 | -91 | -1 |
|  | Location not in atlas | 7 | 5.51 | 22 | -25 | 20 |
|  | R Fusiform Gyrus | 18 | 5.51 | 38 | -79 | -13 |
|  | L IFG (p. Triangularis) | 7 | 5.48 | -53 | 22 | 34 |
|  | Location not in atlas | 5 | 5.40 | -19 | -13 | 18 |
|  | Location not in atlas | 7 | 5.24 | 24 | -85 | 20 |
|  | R MCC | 5 | 5.20 | 4 | -29 | 42 |
|  | L Middle Frontal Gyrus | 5 | 5.13 | -27 | -1 | 56 |
|  | L Inferior Parietal Lobule | 6 | 5.13 | -37 | -35 | 42 |
|  |  |  |  |  |  |  |
| **Block 3** | R Superior Frontal Gyrus | 4004 | 9.35 | 30 | 58 | 20 |
|  | R IFG (p. Orbitalis) | 4004 | 8.72 | 44 | 20 | -5 |
|  | R Middle Frontal Gyrus | 4004 | 8.45 | 38 | 6 | 48 |
|  | Location not in atlas | 296 | 8.58 | 18 | -11 | 22 |
|  | Location not in atlas | 296 | 6.51 | 24 | -31 | 12 |
|  | Location not in atlas | 296 | 6.32 | 16 | 24 | 10 |
|  | R SupraMarginal Gyrus | 2792 | 8.39 | 50 | -43 | 44 |
|  | R Superior Temporal Gyrus | 2792 | 8.22 | 50 | -43 | 16 |
|  | R Angular Gyrus | 2792 | 7.54 | 42 | -63 | 48 |
|  | L IFG (p. Orbitalis) | 453 | 8.27 | -39 | 22 | -1 |
|  | L Putamen | 453 | 6.01 | -27 | 2 | 14 |
|  | L Inferior Parietal Lobule | 1078 | 7.98 | -45 | -57 | 56 |
|  | L SupraMarginal Gyrus | 1078 | 7.43 | -61 | -53 | 38 |
|  | L Inferior Parietal Lobule | 1078 | 6.21 | -47 | -37 | 42 |
|  | L Caudate Nucleus | 44 | 7.73 | -17 | -7 | 22 |
|  | R Putamen | 120 | 7.49 | 30 | -19 | 10 |
|  | R Putamen | 120 | 5.96 | 28 | 2 | 12 |
|  | R Precuneus | 289 | 7.15 | 2 | -63 | 46 |
|  | L IFG (p. Triangularis) | 258 | 7.04 | -47 | 12 | 34 |
|  | L Putamen | 58 | 6.65 | -29 | -23 | 8 |
|  | Location not in atlas | 24 | 6.39 | -17 | -1 | 20 |
|  | R Superior Medial Gyrus | 14 | 6.31 | 4 | 18 | 48 |
|  | L Middle Orbital Gyrus | 192 | 6.26 | -45 | 50 | -1 |
|  | L Middle Frontal Gyrus | 192 | 5.90 | -39 | 52 | 20 |
|  | L Middle Temporal Gyrus | 12 | 6.16 | -61 | -59 | 14 |
|  | R Middle Occipital Gyrus | 24 | 6.16 | 34 | -81 | 32 |
|  | L Middle Frontal Gyrus | 53 | 6.13 | -27 | 2 | 56 |
|  | L Precuneus | 22 | 6.11 | -11 | -65 | 54 |
|  | L Superior Medial Gyrus | 42 | 6.07 | -7 | 38 | 42 |
|  | R Superior Frontal Gyrus | 14 | 6.07 | 16 | 22 | 60 |
|  | R Calcarine Gyrus | 45 | 5.99 | 6 | -81 | 10 |
|  | R Superior Medial Gyrus | 39 | 5.96 | 6 | 36 | 42 |
|  | R ACC | 13 | 5.95 | 10 | 44 | 16 |
|  | L Superior Medial Gyrus | 9 | 5.94 | 2 | 24 | 58 |
|  | Location not in atlas | 8 | 5.90 | 4 | -27 | 28 |
|  | Location not in atlas | 9 | 5.88 | -27 | -33 | 12 |
|  | R Inferior Occipital Gyrus | 26 | 5.87 | 36 | -85 | -9 |
|  | R Middle Frontal Gyrus | 13 | 5.84 | 22 | 54 | 34 |
|  | Location not in atlas | 9 | 5.76 | 20 | -7 | 32 |
|  | R Inferior Occipital Gyrus | 23 | 5.72 | 38 | -81 | 4 |
|  | R Fusiform Gyrus | 22 | 5.67 | 24 | -77 | -7 |
|  | L Middle Temporal Gyrus | 10 | 5.65 | -49 | -53 | 12 |
|  | L Middle Frontal Gyrus | 9 | 5.65 | -31 | 10 | 52 |
|  | L Superior Temporal Gyrus | 24 | 5.63 | -59 | -53 | 20 |
|  | R Middle Temporal Gyrus | 8 | 5.61 | 50 | -77 | 6 |
|  | R Fusiform Gyrus | 6 | 5.60 | 36 | -69 | -13 |
|  | R Superior Temporal Gyrus | 8 | 5.60 | 66 | -35 | 26 |
|  | Location not in atlas | 10 | 5.56 | 44 | -57 | 12 |
|  | L Cuneus | 6 | 5.29 | 2 | -79 | 24 |
|  |  |  |  |  |  |  |
| **Block 4** | R IFG (p. Opercularis) | 1505 | 9.17 | 52 | 14 | 40 |
|  | R IFG (p. Orbitalis) | 1505 | 7.22 | 52 | 22 | 2 |
|  | R Middle Frontal Gyrus | 1505 | 6.75 | 50 | 30 | 26 |
|  | R Inferior Parietal Lobule | 3015 | 8.23 | 50 | -55 | 44 |
|  | R Middle Temporal Gyrus | 3015 | 7.98 | 66 | -41 | 6 |
|  | R Middle Temporal Gyrus | 3015 | 7.81 | 50 | -27 | -5 |
|  | Location not in atlas | 18 | 7.30 | -27 | 14 | 6 |
|  | R Caudate Nucleus | 65 | 7.28 | 16 | -7 | 24 |
|  | R Linual Gyrus | 1916 | 7.24 | 20 | -81 | -9 |
|  | L Fusiform Gyrus | 1916 | 7.02 | -27 | -55 | -9 |
|  | L Cerebelum (VI) | 1916 | 6.83 | -17 | -85 | -11 |
|  | R Middle Orbital Gyrus | 418 | 7.14 | 40 | 54 | -7 |
|  | R Superior Frontal Gyrus | 418 | 7.04 | 34 | 58 | 16 |
|  | R IFG (p. Orbitalis) | 418 | 5.50 | 54 | 34 | -3 |
|  | L IFG (p. Orbitalis) | 139 | 6.85 | -37 | 24 | -5 |
|  | L Inferior Parietal Lobule | 582 | 6.71 | -43 | -63 | 56 |
|  | L SupraMarginal Gyrus | 582 | 6.31 | -57 | -55 | 34 |
|  | R Putamen | 32 | 6.65 | 30 | -21 | 6 |
|  | L Putamen | 14 | 6.52 | -31 | -23 | 4 |
|  | L Precentral Gyrus | 74 | 6.42 | -53 | 8 | 42 |
|  | L Middle Frontal Gyrus | 74 | 5.58 | -43 | 26 | 40 |
|  | R Cerebelum (VI) | 23 | 6.41 | 10 | -71 | -15 |
|  | R Precuneus | 138 | 6.39 | 10 | -53 | 52 |
|  | R Superior Medial Gyrus | 42 | 6.25 | 12 | 52 | 44 |
|  | R Superior Medial Gyrus | 49 | 6.23 | 4 | 36 | 46 |
|  | R Fusiform Gyrus | 31 | 6.15 | 30 | -57 | -5 |
|  | L Caudate Nucleus | 5 | 6.06 | -17 | -7 | 22 |
|  | R ACC | 14 | 6.03 | 8 | 46 | 14 |
|  | L Middle Temporal Gyrus | 25 | 5.99 | -57 | -51 | 8 |
|  | L Middle Occipital Gyrus | 12 | 5.96 | -39 | -77 | 20 |
|  | Location not in atlas | 6 | 5.95 | -47 | 44 | -7 |
|  | Location not in atlas | 26 | 5.87 | 32 | -3 | -11 |
|  | R Calcarine Gyrus | 50 | 5.86 | 20 | -87 | 22 |
|  | Location not in atlas | 16 | 5.86 | -17 | 4 | 16 |
|  | R ACC | 9 | 5.83 | 6 | 42 | 30 |
|  | R Putamen | 8 | 5.78 | 24 | 2 | 14 |
|  | Location not in atlas | 10 | 5.74 | -21 | -21 | 22 |
|  | Location not in atlas | 12 | 5.73 | -5 | -35 | -11 |
|  | L Middle Temporal Gyrus | 9 | 5.72 | -55 | -45 | -1 |
|  | R Superior Medial Gyrus | 9 | 5.72 | 18 | 64 | 18 |
|  | R Caudate Nucleus | 8 | 5.70 | 14 | 16 | 10 |
|  | L Precentral Gyrus | 14 | 5.70 | -39 | -15 | 44 |
|  | L Precuneus | 16 | 5.69 | -9 | -75 | 46 |
|  | L Middle Occipital Gyrus | 81 | 5.66 | -31 | -81 | 24 |
|  | L Inferior Parietal Lobule | 20 | 5.66 | -31 | -57 | 50 |
|  | L Middle Temporal Gyrus | 9 | 5.64 | -65 | -51 | 14 |
|  | R Superior Medial Gyrus | 18 | 5.63 | 12 | 48 | 8 |
|  | L Middle Temporal Gyrus | 6 | 5.59 | -65 | -53 | 6 |
|  | L Precuneus | 21 | 5.56 | -9 | -57 | 52 |
|  | L MCC | 8 | 5.54 | -1 | -51 | 56 |
|  | Location not in atlas | 7 | 5.50 | -31 | -47 | 40 |
|  | Location not in atlas | 9 | 5.48 | -41 | -33 | 38 |
|  | R Middle Temporal Gyrus | 8 | 5.47 | 54 | -3 | -23 |
|  | L Middle Temporal Gyrus | 11 | 5.46 | -55 | -27 | -1 |
|  | Location not in atlas | 7 | 5.45 | -27 | -33 | 14 |
|  | L Precuneus | 7 | 5.44 | -17 | -77 | 36 |
|  | L Caudate Nucleus | 10 | 5.41 | -19 | 22 | 10 |
|  | L Superior Medial Gyrus | 12 | 5.40 | -7 | 36 | 42 |
|  | L Inferior Parietal Lobule | 8 | 5.38 | -41 | -53 | 42 |
|  | L Cuneus | 7 | 5.36 | -9 | -83 | 44 |
|  | L Precuneus | 6 | 5.33 | -7 | -71 | 52 |
|  | R Middle Temporal Gyrus | 5 | 5.25 | 44 | -77 | 6 |

**Supplementary Table 5**. This table presents voxel-wise whole-brain brain activation patterns elicited by the unconditioned stimulus (US) during the fear conditioning task. Results are FWE corrected (*p* < 0.05)

|  |  |  | MNI Coordinates | | |
| --- | --- | --- | --- | --- | --- |
| Brain region | Cluster size | t-values | x | y | z |
| R Linual Gyrus | 1480 | 13.75 | 16 | -87 | -7 |
| L Cerebelum (VI) | 1480 | 12.44 | -9 | -85 | -9 |
| Location not in atlas | 1480 | 9.77 | 34 | -67 | 4 |
| R Insula Lobe | 2990 | 13.23 | 40 | -17 | 18 |
| R Insula Lobe | 2990 | 13.22 | 42 | -1 | -7 |
| R SupraMarginal Gyrus | 2990 | 10.74 | 62 | -37 | 46 |
| Location not in atlas | 559 | 12.68 | -41 | -13 | -9 |
| L Insula Lobe | 559 | 8.99 | -43 | 8 | -5 |
| L Insula Lobe | 559 | 6.89 | -39 | -21 | 12 |
| R IFG (p. Triangularis) | 452 | 12.63 | 48 | 44 | 6 |
| Cerebellar Vermis (10) | 970 | 12.38 | -1 | -55 | -23 |
| Location not in atlas | 970 | 9.36 | -21 | -49 | -25 |
| Location not in atlas | 970 | 6.01 | 18 | -43 | -23 |
| L IFG (p. Triangularis) | 737 | 11.86 | -53 | 34 | 6 |
| L IFG (p. Orbitalis) | 737 | 6.43 | -39 | 40 | -9 |
| Location not in atlas | 1244 | 9.10 | -61 | -47 | 48 |
| L Middle Temporal Gyrus | 1244 | 9.09 | -63 | -59 | 2 |
| L Angular Gyrus | 1244 | 8.32 | -51 | -69 | 32 |
| L Superior Medial Gyrus | 903 | 8.98 | -5 | 54 | 38 |
| R Superior Frontal Gyrus | 903 | 7.54 | 16 | 58 | 32 |
| L Superior Medial Gyrus | 903 | 7.17 | -7 | 64 | 18 |
| Location not in atlas | 51 | 8.63 | -33 | -71 | 4 |
| R MCC | 84 | 7.97 | 2 | -17 | 36 |
| L PCC | 218 | 7.90 | -5 | -47 | 36 |
| L Insula Lobe | 56 | 7.60 | -35 | 6 | 14 |
| Location not in atlas | 127 | 7.19 | -23 | -23 | 26 |
| R Postcentral Gyrus | 147 | 7.15 | 50 | -11 | 38 |
| R Amygdala | 12 | 6.72 | 26 | -9 | -13 |
| R Middle Temporal Gyrus | 44 | 6.67 | 66 | -33 | 2 |
| L Precentral Gyrus | 101 | 6.53 | -47 | -15 | 36 |
| L ACC | 10 | 6.51 | -3 | 26 | 26 |
| L Middle Frontal Gyrus | 58 | 6.05 | -29 | 24 | 50 |
| R Middle Temporal Gyrus | 14 | 5.88 | 60 | -57 | 2 |
| L Caudate Nucleus | 6 | 5.82 | -15 | 8 | 18 |
| R Middle Temporal Gyrus | 8 | 5.63 | 54 | -35 | -1 |
| L Middle Frontal Gyrus | 18 | 5.58 | -39 | 10 | 52 |
| L Superior Temporal Gyrus | 5 | 5.42 | -63 | -29 | 14 |
| R Angular Gyrus | 8 | 5.41 | 58 | -63 | 34 |
| R Superior Medial Gyrus | 13 | 5.40 | 14 | 38 | 52 |
| L Superior Medial Gyrus | 5 | 5.18 | -11 | 48 | 10 |

**Supplementary Figure 1**. Experimental design. Two virtual characters served as conditioned stimuli (CS). One of the virtual characters served as fear cue (CS+) and predicted the unconditioned stimulus (US) whereas the other character served as safety cue (CS-) and was never associated with the US. Each CS-type appeared 16 times each for 6s with an inter-stimulus interval of 8-12s. Eight of the CS+ presentations co-terminated with presentation of the US (50% reinforcement schedule). Four stimulus presentation orders were used to counterbalance CS across subjects. Prior to the experiment, participants were told that they could learn to predict the US but were not told which character served as CS+.
